# Notfallmedizin in der Deutschen Gesellschaft zur Rettung Schiffbrüchiger – Auswertung medizinischer Notfälle auf der Nord- und Ostsee über 2 Jahre

**DOI:** 10.1007/s00101-020-00885-5

**Published:** 2020-11-24

**Authors:** S. Schemke, H. Schwalbe, L. Grunewald, H. Maurer

**Affiliations:** 1 Universität zu Lübeck, Klinik für Anästhesiologie, Lübeck, Deutschland; 2 Deutsche Gesellschaft zur Rettung Schiffbrüchiger (DGzRS), Bremen, Deutschland; 3 Universitätsklinikum Schleswig-Holstein, Campus Lübeck, Klinik für Anästhesiologie und Intensivmedizin, Ratzeburger Allee 160, 23562 Lübeck, Deutschland

**Keywords:** Seenotrettung, Maritime Medizin, SAR, Reanimation, Hypothermie, Sea rescue, Maritime medicine, SAR—Search and Rescue, Resuscitation, Hypothermia

## Abstract

**Hintergrund:**

Die logistischen Besonderheiten eines maritimen Notfallortes und die häufig zusätzlich drohende akzidentelle Hypothermie machen die Versorgung medizinischer Notfälle auf dem Meer besonders anspruchsvoll. In dieser Arbeit sollen die Charakteristika notfallmedizinischer Einsätze der Deutschen Gesellschaft zur Rettung Schiffbrüchiger (DGzRS) als Hauptträgerin der nichthelikopterbasierten medizinischen Seenotrettung auf den Meeren vor der deutschen Küste beschrieben werden.

**Material und Methoden:**

Es erfolgte eine retrospektive Analyse aller Einsätze der DGzRS der Jahre 2017 und 2018. Die Einsatzdaten und –zeiten sowie die Erkrankungsschwere der Patienten (graduiert mittels NACA-Score) wurden ausgewertet und exemplarisch mit denen eines NEF der Hansestadt Lübeck verglichen.

**Ergebnisse:**

Bei insgesamt 182 medizinischen Einsätzen wurden 224 Patienten behandelt. Die Einsatzeinheiten der DGzRS benötigten im Mittel 30 ± 21 min bis zur Ankunft und 43 ± 30 min für Rettung, Behandlung und Transport. Bei 63 Einsätzen wurden die Patienten durch einen Notarzt betreut, der bei 44 Einsätzen durch die Landrettung herangeführt wurde. Durch die Wartezeit auf bordfremdes Personal wurde bei 26 Einsätzen die Abfahrt um im Mittel 18 ± 7 min verzögert.

Die durchschnittliche Erkrankungsschwere in der Seenotrettung war signifikant höher als im Lübecker Notarztdienst; es gab vergleichbar häufig Reanimationen und Todesfälle.

**Schlussfolgerung:**

Trotz der hohen Krankheitsschwere medizinischer Notfälle auf den Meeren vor Deutschlands Küste treffen Notärzte dort häufig mit erheblicher Verzögerung ein. Es gibt den dringenden Bedarf effektiverer Unterstützung der DGzRS durch für den maritimen Einsatz ausgebildetes ärztliches Personal.

## Abstract (en)

**Background:**

The logistic peculiarities of an emergency maritime location and the frequent additional threat of accidental hypothermia mean that the treatment of medical emergencies at sea are particularly demanding. This article describes the characteristics of emergency medical missions of the German Maritime Search and Rescue Service (DGzRS) as the main provider of non-helicopter-based medical maritime rescue on the seas off the coasts of Germany.

**Material and methods:**

A retrospective analysis of all missions by the DGzRS in 2017 and 2018 was carried out. The data and times of the missions as well as the severity of the diseases of the patients (graduated using the NACA score) were evaluated and exemplarily compared to those of a medical emergency ambulance service from the City of Lübeck.

**Results:**

In a total of 182 medical missions 224 patients were treated. The mission units of the DGzRS needed a mean time of 30 ± 21 min up to arrival and 43 ± 30 min for rescue, treatment and transport. In 63 missions the patients were accompanied by an emergency physician, who was brought in from the ground rescue service in 44 missions. Due to the waiting time for boarding of the additional personnel, the departure in 26 missions was delayed by an average of 18 ± 7 min.

The average severity of the disease in the maritime rescue was significantly higher than in the emergency medical service of Lübeck but the number of resuscitations and fatalities were comparable.

**Conclusion:**

Although the severity of medical emergencies on the seas off the coasts of Germany was high, the emergency physicians frequently arrived with a considerable delay. There is an urgent need for an effective support of the DGzRS by medical personnel specifically trained for maritime missions.

## Einleitung

Auf den Meeren vor Deutschlands Küsten befindet sich zu jedem Zeitpunkt zu privaten oder beruflichen Zwecken eine große Anzahl von Menschen. Ebenso wie an Land kommt es bei diesen Personengruppen zu medizinischen Problemen vom internistischen Notfall bis zum schweren Arbeitsunfall. Die logistischen Besonderheiten des Notfallortes, wie lange Fahrzeiten, der komplizierte Transfer von Mensch und Material zwischen wasser-, luft- und bodengebundenen Fahrzeugen, oder die potenziell zusätzlich drohende akzidentelle Hypothermie aufgrund der klimatischen Besonderheiten auf See machen diese Notfälle besonders anspruchsvoll.

## Thematischer Hintergrund

Die Versorgung medizinischer Notfälle auf den Meeren vor Deutschlands Küste wird aktuell durch eine Kombination verschiedener Organisationen erbracht. In unmittelbarer Nähe zur Küste stehen z. B. die Deutsche Lebens-Rettungs-Gesellschaft e. V. (DLRG) oder die Wasserwacht bereit. Ist ein Schiff beteiligt, wird häufig die Unterstützung der Deutschen Gesellschaft zur Rettung Schiffbrüchiger (DGzRS) nötig. In komplexen Schadenslagen besteht weiterhin die Möglichkeit einer Hilfeleistung durch das Havariekommando [4] mit den jeweiligen Feuerwehren oder das Heranführen des Landrettungsdienstes über alternative Seefahrzeuge.

Ereignet sich der medizinische Notfall nicht im unmittelbaren Küstengebiet, werden Schiffe größeren Ausmaßes oder Helikopter notwendig. Hier steht die Flotte der DGzRS, ergänzt durch die „offshore“-tauglichen Rettungshubschrauber Deutschlands oder benachbarter Küstenländer, zur Verfügung. Zu beachten ist hierbei, dass aufgrund der besonderen Bedingungen auf See das Einsatzmittel Helikopter nicht in jedem Falle nutzbar und seine maximale Flugdauer (abhängig von z. B. Wind und Beladung; Reichweite der Sea King: ca. 1500 km Gesamtstrecke [11]) beschränkt ist. Dunkelheit, Nebel, Sturm oder Besonderheiten wie Wirbelschleppen leeseitig angeflogener Windkraftanlagen [5, 15] oder die unzureichende technische Ausstattung für den Transfer zwischen Schiff und Helikopter können einen Einsatz je nach Hubschraubertyp einschränken oder unmöglich machen.

Ein großer Teil der medizinischen Notfälle auf See kann und wird nur unter Zuhilfenahme der Flotte der DGzRS adäquat versorgt werden. Die flächendeckende Einsatzbereitschaft der durch die DGzRS betriebenen 20 Seenotrettungskreuzer (>20 m, hauptamtlich rund um die Uhr besetzt) und 39 Seenotrettungsboote (ehrenamtlich durch Rufbereitschaften besetzt) zu jeder Uhrzeit und an jedem Tag des Jahres zu realisieren, erfordert aufgrund der hohen Zahl an notwendigen leistungsstarken Wasserfahrzeugen und des großen Bedarfs adäquat geschulter und kurzfristig verfügbarer Besatzungsmitglieder einen immensen Material- und Zeitaufwand. Die DGzRS nimmt dafür keine öffentlichen Gelder oder Steuern in Anspruch.

Bei der Betrachtung der Notfallversorgung muss bedacht werden, dass der medizinische Ausbildungsgrad der Seenotretter sehr heterogen ist; ein erweitertes Ersthelferniveau liegt hierbei in jedem Fall vor. Die Vorhaltung medizinischer Hilfe auf (not)ärztlichem Niveau ist nicht Bestandteil des Aufgabengebiets der DGzRS.

Die vorliegende Arbeit soll durch Analyse aller Einsätze mit medizinischem Hintergrund der Seenotrettungsboote und -kreuzer in den deutschen Gebieten von Nord- und Ostsee helfen, die Häufigkeit solcher medizinischen Notfälle genauer zu beschreiben. Weiterhin soll sie erste Anhaltspunkte dafür liefern, welche Akuität und Erkrankungsschwere vorliegen, welche Art von medizinischem Fachpersonal diese Notfälle versorgt und wie lang die dafür notwendigen Einsatzzeiten sind.

## Methoden

Die Datenerhebung erfolgte durch Analyse des DGzRS-internen Datendokumentationssystems. In diesem wird intranetbasiert jede Fahrt eines DGzRS-Einsatzmittels durch ein Besatzungsmitglied digital dokumentiert. (Die internen Einsatzprotokolle orientieren sich an den DIVI-Empfehlungen und entsprechen weitestgehend dem typischen Aufbau eines im Landrettungsdienst genutzten Protokolls). Die jeweiligen Einsatzprotokolle wurden gesichtet und bei medizinischer Relevanz in die Studie eingeschlossen. Aufgrund der Aussagekraft bezüglich drohender Lebensgefahr und zu erwartender Morbidität und Mortalität von Unfallopfern [17] wurden die betrachteten Patienten mit dem NACA-Index kategorisiert. Der angegebene NACA-Score wurde, wo auf Basis der vorliegenden Daten möglich, von den Autoren (einem Assistenzarzt für Anästhesie mit der Zusatzbezeichnung Notfallmedizin sowie einem Facharzt mit den Zusatzbezeichnungen Notfallmedizin und Intensivmedizin mit langjähriger Berufserfahrung) nachträglich vergeben. Für die Eingruppierung wurden auch andernorts veröffentlichte Kriterien [12] zurate gezogen. Reanimationen und Todesfälle wurden gesondert betrachtet. Exemplarische Zuordnungen von Einsätzen zu NACA-Scores finden sich in Tab. 1.

**Table 1:** 

NACA I	Patient mit umfangreicher Kälteschutzkleidung nach Havarie im Wasser, folgend Eigenrettung an Land ohne Hypothermie
NACA II	Fingerfraktur eines Kindes an Bord eines Freizeitbootes; Hyperventilation an Bord einer Fähre
NACA III	Torsionstrauma des Knies an Bord mit mechanischer Blockade der Extension/Flexion; schmerzhafte Nierenkolik an Bord eines Segelboots
NACA IV	Schlaganfall auf einem Kreuzfahrtschiff; Rettung dreier unterkühlter Wattwanderer, bereits bis zum Hals unter Wasser
NACA V	STEMI auf Fischerboot; Patient nach Havarie an Leine hinter Boot im Wasser ohne Kältezittern mit reduzierter Vigilanz und Bradykardie
NACA VI	Reanimation
NACA VII	Todesfall

Die Graduierung einer akzidentellen Hypothermie erfolgte nach dem „Swiss staging system“ [14], soweit gemessen an der Temperatur, ansonsten anhand der klinischen Symptomatik (Tab. 2).

**Table 2:** 

Hypothermiegrad	Symptomatik	Körperkerntemperatur (°C)
Grad I	Bewusstsein uneingeschränkt, Kältezittern	35–32
Grad II	Bewusstseinseinschränkung, kein Kältezittern	32–28
Grad III	Bewusstlos, kein Kältezittern, Lebenszeichen vorhanden	28–24
Grad IV	Keine Lebenszeichen	<24

Die in der Untersuchung verwendeten exemplarischen Vergleichswerte eines Notarztdienstes wurden auf dem am Universitätsklinikum Schleswig-Holstein, Campus Lübeck, stationierten NEF des Rettungsdienstes der Hansestadt Lübeck erhoben und repräsentieren das gesamte Einsatzspektrum eines rund um die Uhr verfügbaren NEF der Stadtrettung einer deutschen Großstadt (Lübeck: ca. 215.000 Einwohner).

Die statistische Aufarbeitung erfolgte mithilfe des Programmes MedCalc (MedCalc Software bvba, Acacialaan 22, 8400 Ostend, Belgien). Die grafische Darstellung der Ergebnisse erfolgte mit Microsoft Excel 2013 (Microsoft Corporation, One Microsoft Way, Redmond, WA, USA). Bei punktuell unvollständigen Daten wurden lediglich die vorhandenen Werte als Datenbasis genutzt. Bei der Betrachtung von Einsatzzeiten wurde ein Mittelwert berechnet und der dazugehörige Standardfehler angegeben. Weiterhin wurden der Median der Erkrankungsschwere (NACA-Score) berechnet und als Maß der Streubreite das erste und dritte Quartil angegeben (Q1; Q3). Statistische Signifikanz wurde mithilfe des Mann-Whitney-Tests bzw. des zweiseitigen Chi-Quadrat-Tests errechnet. Bei einer Irrtumswahrscheinlichkeit unter 5 % (*p* < 0,05) wurden die beobachteten Unterschiede als signifikant erachtet.

Aufgrund der rein retrospektiven ausschließlichen Analyse von Alter und Geschlecht in Bezug auf personenbezogene Daten konnte auf ein Ethikvotum verzichtet werden.

## Ergebnisse

Der weit überwiegende Teil der 4212 (2017: 2056; 2018: 2156) dokumentierten Einsätze der DGzRS in den Jahren 2017 und 2018 galt nichtmedizinischen Problemen (vornehmlich Hilfeleistung bei technischen Schwierigkeiten). In den Jahren 2017 und 2018 wurden 182 Einsätze als medizinisch relevante primäre Einsatzfahrten erkannt (2017: 103; 2018: 79). Zusätzlich bearbeitete die DGzRS in diesen beiden Jahren noch ungefähr 669 Krankentransporte von den Inseln zum Festland (2017: 374; 2018: 295), wobei diese Zahl retrospektiv nicht exakt zu ermitteln ist [8]. Die medizinische Verantwortung bei den Krankentransporten obliegt dabei der begleitenden medizinischen Crew des kommunalen Trägers des Rettungsdienstes oder der Klinik.

### Einsatzstruktur und nautische Bedingungen

**Figure 1:**
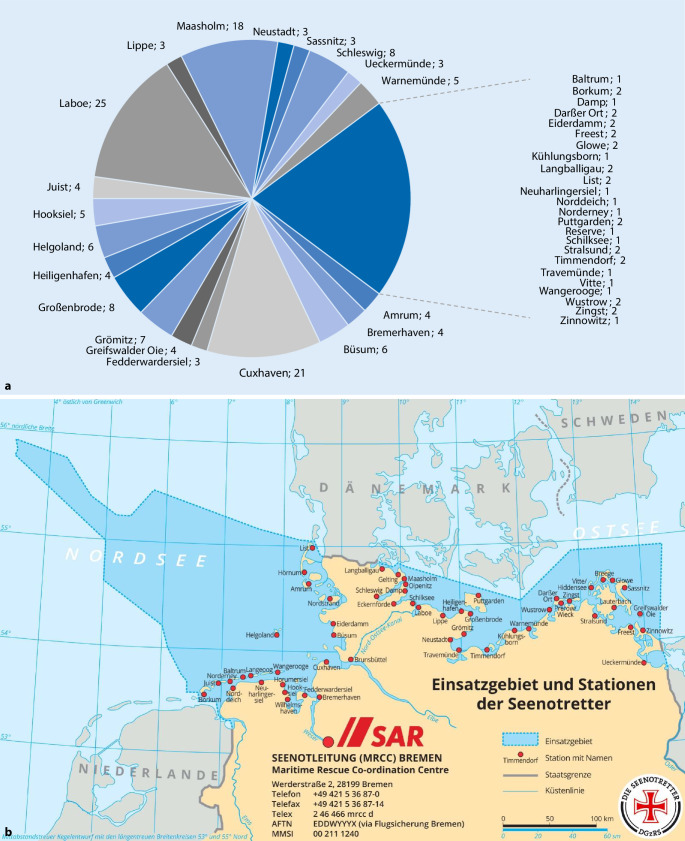

Von 181 dahingehend analysierbaren medizinisch relevanten Einsätzen entfielen 122 Einsatzfahrten auf durch hauptamtlich angestellte Seenotretter besetzte Seenotrettungskreuzer und 59 Einsatzfahrten auf durch ehrenamtlich tätige Seenotretter besetzte Seenotrettungsboote der DGzRS. Hier zeigten sich große Unterschiede in der Einsatzhäufigkeit zwischen den einzelnen Einsatzmitteln (Abb. 1).

Die Wetterbedingungen zum Zeitpunkt der medizinischen Einsätze der DGzRS waren zu 52 % sonnig, zu 37 % bedeckt, zu 8 % regnerisch, zu 4 % trüb und bei 1 % der Einsätze schneite es. Die durchschnittliche Windstärke im Einsatz betrug 4 ± 1 Beaufort (4 Beaufort entsprechen 20–28 km/h) bei durchschnittlich 0,7 ± 0,4 m Wellenhöhe und im Mittel 16 ± 4 km Sichtweite. Die durchschnittliche Wassertemperatur während der medizinischen Einsätze betrug 15 ± 5 °C. Pro Einsatz wurden im Mittel 11 ± 10 Seemeilen (1 Seemeile = 1852 m) zurückgelegt.

### Einsatzzeiten

Vom Ablegen des Einsatzmittels bis zur Ankunft am Patienten vergingen im Mittel 30 ± 21 min. Hierbei hatten die Seenotrettungskreuzer tendenziell längere Anfahrtsdauern (Seenotrettungskreuzer 35 ± 26 min vs. Seenotrettungsboote 21 ± 10 min). Nach Ankunft am Patienten benötigten die Rettungseinheiten der DGzRS im Mittel 43 ± 30 min für Rettung, Behandlung und Transport der Patienten (Seenotrettungskreuzer 48 ± 35 min vs. Seenotrettungsboote 32 ± 21 min). Die mittlere Dauer medizinischer Einsätze betrug 1:40 ± 1:04 h (Seenotrettungskreuzer 1:46 ± 1:12 h vs. Seenotrettungsboote 1:26 ± 0:46 h). Konnte die auf Basis des Hilferufs als notwendig erachtete medizinische Besatzung nicht durch die Seenotrettungseinheit selbst gestellt oder über parallele Wege herangeführt werden, musste mit dem Ablegen auf eintreffendes medizinisches Personal des Landrettungsdienstes oder besondere Spezialkräfte (Taucher oder Höhenretter) gewartet werden. Dies führte bei 26 Einsätzen zu einer Verzögerung des Ablegens von im Mittel 18 ± 7 min (Wartedauer minimal 4 min und maximal 40 min).

### Medizinisches Einsatzspektrum

**Figure 2:**
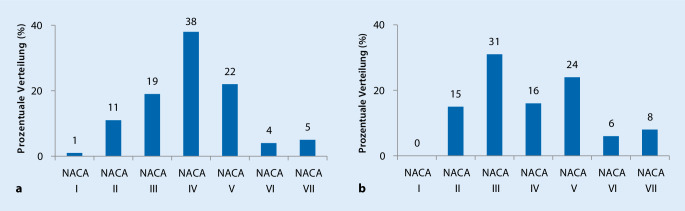

**Figure 3:**
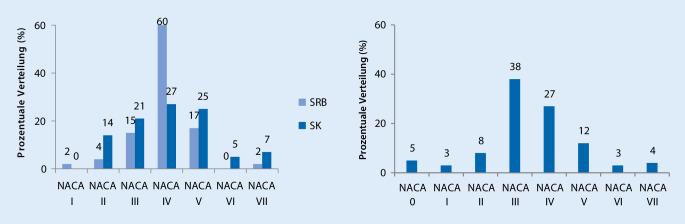

Im Rahmen der 182 medizinischen Einsätze waren insgesamt 224 Personen betroffen. Diese waren im Mittel 46 ± 16 Jahre alt (Durchschnittsalter im Notarztdienst der Hansestadt Lübeck: 61 Jahre) und zu 33 % weiblich bzw. 67 % männlich. Der Median der NACA-Kategorisierung lag in der Seenotrettung sowohl mit als auch ohne Berücksichtigung von aus dem Wasser geretteten Patienten in der Gesamtbetrachtung und in der isolierten Betrachtung der Seenotrettungsboote und Seenotrettungskreuzer bei 4 (Abb. 2). In der Vergleichsregion des Lübecker Notarztdienstes fanden sich verhältnismäßig weniger NACA-IV- und NACA-V-kategorisierte Patienten. Der Schwerpunkt lag hier in der Kategorie NACA III und somit außerhalb akuter Lebensgefahr (Abb. 3). Der Median der NACA-Kategorisierung lag im Lübecker Notarztdienst bei 3 (3; 4).

Die Erkrankungsschwere unterschied sich sowohl im Vergleich zwischen allen Einsätzen der DGzRS und denen des Lübecker Notarztdienstes (*p* < 0,0001), als auch den Einsätzen der DGzRS ohne Betrachtung von Patienten im Wasser und denen des Lübecker Notarztdienstes (*p* = 0,0018).

**Figure 4:**
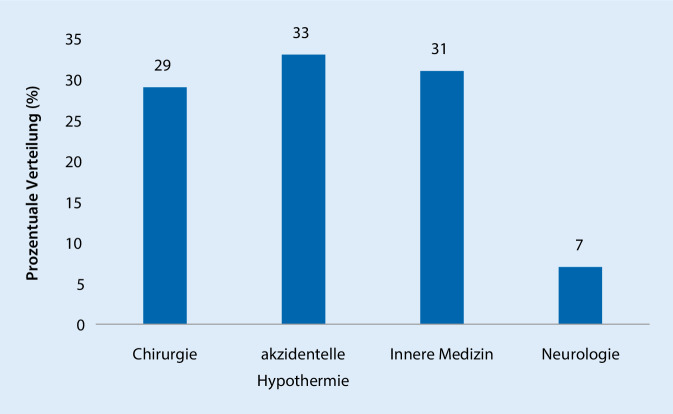

In den Jahren 2017/2018 wurden 102 Patienten aus dem Wasser gerettet, von diesen wiesen 63 eine akzidentelle Hypothermie auf (90 % Hypothermie I, 7 % Hypothermie II, 3 % Hypothermie III; bei Reanimationen war retrospektiv keine sichere Beurteilung möglich, ob eine Hypothermie die primäre Todesursache war). Darüber hinaus wurde die Hilfe der DGzRS vergleichbar häufig aufgrund von internistischen oder chirurgischen Problemen (chirurgische Indikationen: 81 % traumatisch, davon 98 % Monotrauma und 2 % Polytrauma; 19 % nicht traumatisch) nötig. Neurologische Erkrankungen waren als primäre Einsatzindikation (7 %) am seltensten ausschlaggebend (Abb. 4). Die der Einsatzindikation zuzuordnende fachliche Gruppierung unterscheidet sich bei der DGzRS deutlich von der des Lübecker Notarztdienstes. In Lübeck lagen zu 74 % internistische Problematiken vor; die akzidentelle Hypothermie war mit unter 1 % deutlich seltener.

Die Seenotretter mussten 2017 und 2018 insgesamt bei 10 Patienten Reanimationsmaßnahmen durchführen (5,5 %). Im Lübecker Notarztdienst mussten bei 6779 Einsätzen 297 Reanimationen durchgeführt werden (4,4 %). Insgesamt waren in den Einsätzen der DGzRS in den beiden Jahren, frustrane Sucheinsätze ausgenommen, 9 tote Patienten zu beklagen (4,9 %). Im Rahmen des Notarztdienstes der Stadt Lübeck waren 242 tote Patienten zu beklagen (3,6 %).

**Figure 5:**
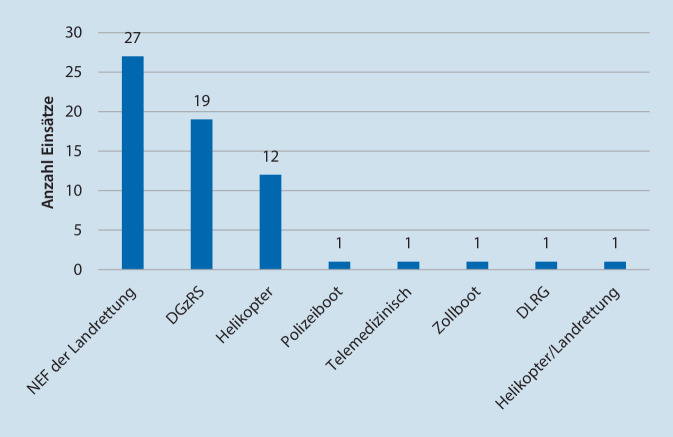

In insgesamt 63 Einsätzen (34,6 % der medizinisch relevanten Einsätze) wurde/wurden der oder die Patienten durch einen Notarzt betreut und dies dokumentiert. Bei diesen 63 Notarzteinsätzen konnte in 19 Fällen ein freiwilliger Seenotarzt der DGzRS eingesetzt werden, und in 44 Fällen wurde ein externer Notarzt aufgenommen (Abb. 5), welcher in den meisten Fällen mittels Landrettungsmitteln vor Ablegen des Bootes an Bord kam.

Insgesamt wurde auf mindestens 31 Einsatzfahrten eine Aufnahme weiterer, nichtärztlicher Einsatzkräfte nötig (25-mal RTW-Besatzung, 3‑mal Höhenretter, einmal Polizei, einmal Feuerwehrtaucher, einmal Feuerwehrmänner). In 18 Einsätzen, die durch die DGzRS abgearbeitet wurden, wurde dokumentiert, dass ein Helikopter beteiligt war. Der am häufigsten bei medizinischen Einsätzen in Zusammenarbeit mit der DGzRS eingesetzte Helikopter war der für die notfallmedizinische Versorgung von Offshorewindparks zuständige und in Sankt Peter-Ording stationierte Helikopter „Northern Rescue 01“.

In 126 Einsätzen wurde der Übergabepartner dokumentiert. In 64 % der Fälle wurden die Patienten an einen RTW übergeben, in 24 % der Fälle an RTW und Notarzt. In 6 % der Fälle wurde der Patient durch den RTH und in 2 % der Fälle durch ein anderes Rettungsmittel der DGzRS transportiert (was nicht als eigenständiger Einsatz gewertet wurde). In je 1 % der Fälle wurden die Patienten an einen KTW, die DLRG, die Polizei oder ein Taxi übergeben.

## Diskussion

Naturgemäß sind Rettungseinsätze auf See vom abtreibenden Kitesurfer über den Herzinfarkt auf dem Kreuzfahrtschiff, den Arbeitsunfall auf dem Frachtschiff oder Fischkutter und das Polytrauma auf der Segeljacht bis zur Reanimation im Watt sehr heterogen.

Die wichtigste Voraussetzung zur möglichst schnellen Hilfeleistung bei allen maritimen Notfällen ist erfahrenes seemännisches Vorgehen. Die Rettungseinheit muss den Patienten rund um die Uhr bei jedem Wetter schnell erreichen, das medizinische Team zu diesem überwechseln, ihn versorgen, auf das Rettungsschiff bringen und schnell einer definitiven Versorgung zuführen. Die alltäglichen Umstände stellen hierbei hohe Ansprüche an die Mannschaft. Deshalb besetzt die DGzRS ihre Seenotrettungskreuzer durch fest angestellte und entlohnte, oder im Falle der Seenotrettungsboote ehrenamtlich aktive seemännisch geschulte Rettungsmänner und -frauen mit medizinischer Kenntnis, mindestens auf erweitertem Ersthelferniveau (dem sog. SAR-Ersthelfer) [3].

Auch medizinisch sind Seenotrettungseinsätze überdurchschnittlich herausfordernd. Oft müssen schwer kranke Patienten unter widrigsten Umständen und mit geringeren Ressourcen, als aus der klinischen Praxis gewohnt, versorgt werden. Der medizinische Teil des Behandlungsteams, wenn nicht in Form von Seenotrettern mit umfangreicherer medizinischer Vorausbildung bereits ausreichend vorhanden, wird durch freiwillige Seenotärzte ergänzt, die durch interne Fortbildungen der DGzRS auf die Besonderheiten der Arbeit auf See vorbereitet werden. Da dies ausschließlich ehrenamtlich erfolgt, steht regelmäßig keine arztbesetzte Rettungseinheit zur Verfügung. Hier wird dann versucht, Kräfte aus der umliegenden Regelrettung hinzuzuziehen.

Die Vielfältigkeit möglicher Einsätze in Kombination mit einem variabel zusammengesetzten Rettungsteam, macht bereits die Dringlichkeit einer Analyse der anfallenden Aufgaben und der dafür zur Verfügung stehenden Ressourcen der deutschen Seenotretter deutlich.

Die interne Dokumentation ist geeignet, die geleisteten Einsätze zu erfassen und daraus auch retrospektiv die medizinisch relevanten zu erkennen und zu analysieren. Die Möglichkeiten der Dokumentation eines Einsatzes sind hierbei denen des Notarzteinsatzes in der Landrettung gleichwertig. Häufig wurden diese durch die Besatzung aber nicht vollständig ausgeschöpft. Als erstes Ergebnis der Untersuchung sollen die Dokumentationsqualität und konsequente Nutzung der vorhandenen Dokumentationsmöglichkeiten durch die Besatzung in den folgenden Jahren verbessert werden, um so in Zukunft belastbarere Informationen über die medizinischen Einsätze der deutschen Seenotretter zu erhalten. Die uneinheitliche Dokumentation von medizinischen Notfällen ist im maritimen Umfeld ein bereits in vorigen Untersuchungen beschriebenes Problem [6, 15].

Der Großteil der Rettungseinsätze findet in den Sommermonaten und unter verhältnismäßig ruhigen Wetterbedingungen statt. Trotz der häufig nichtwinterlichen Bedingungen (durchschnittliche Wassertemperatur zum Einsatzzeitpunkt 15 ± 5 °C) ist zu beachten, dass auch solche Wassertemperaturen lebensbedrohlich sein können und insbesondere bei 15 °C und darunter der unerwartete Sturz ohne Schutzkleidung ins Wasser lebensgefährlich ist [1]. Die wenigsten hypothermen Patienten der DGzRS trugen optimal isolierende Überlebensanzüge (Verlängerung der Überlebenszeit in 1 °C kaltem Wasser auf bis zu >1 Tag [9]). Ein leicht bekleideter Mensch verliert in 0 °C kaltem Wasser ca. 6 °C/h und überlebt damit wahrscheinlich im Mittel 1–1,5 h [10], wenn er vor der Unterkühlung nicht bereits ertrinkt (insbesondere bei den durch die DGzRS häufig angetroffenen Freizeitsportlern mit Sturz ins Wasser, Abtreiben und erschöpfenden frustranen Rettungsbemühungen besteht hier große Gefahr des Ertrinkens während der Anfahrt der Seenotrettungseinheit). In führend hypothermen Patienten besteht bei richtiger Rettung und Behandlung eine große Wahrscheinlichkeit der erfolgreichen Therapie, was eine zügige und gut organisierte Hilfeleistung in diesen Fällen besonders wichtig macht [13].

Zum Windpark BARD Offshore 1 beispielsweise (ca. 100 km ab Küstenlinie) betrug die mittlere Eintreffzeit eines Rettungshubschraubers einer Studie mit 39 Einsätzen zufolge 107 min [7]. Die Ankunftsdauer schiffsbasierter medizinischer Hilfe an solchen Einsatzstellen dürfte länger sein.

Die durch die DGzRS durchgeführten Einsätze erforderten eine Anfahrtsstrecke von im Mittel 11 ± 10 Seemeilen. Die mittlere Gesamtdauer medizinischer Einsatzfahrten der DGzRS betrug 1:40 ± 1:04 h. Hier wird die Bedeutung leistungsstarker Spezialschiffe klar, die den jeweiligen Einsatzort auch unter ungünstigen Umgebungsbedingungen möglichst schnell erreichen und verlassen können.

Im Mittel benötigten die Rettungsmittel 30 ± 21 min bis zur Ankunft am Patienten. Beachtet werden muss, dass in vielen Einsatzlagen die DGzRS-Einheit das einzige geeignete Rettungsmittel darstellt. Auch zeitkritische Krankheitsbilder wie Schlaganfall oder Herzinfarkt können also u. U. erst binnen dieser Frist erreicht werden. Hier besteht ein deutlicher Unterschied zu den Hilfsfristen im Landrettungsdienst. Hinzu kommt der erhöhte Zeitbedarf für den Transport des Patienten an Land.

Bei den Seenotrettungseinsätzen wird regelmäßig das Aufnehmen weiteren Personals nötig. Im Großteil der Einsätze war das zusätzlich notwendige Personal ein Arzt. Von den insgesamt 63 arztbegleiteten Einsätzen konnte in 19 Einsätzen ein ehrenamtlich tätiger Arzt der DGzRS eingesetzt werden. In 44 Fällen wurde ein externer Notarzt gerufen. Das Heranführen externer Kräfte (meist Notärzte) erfolgte in den meisten Fällen durch den Regelrettungsdienst. Dies führte in 26 Einsätzen (14 % aller Einsätze) zu einer dokumentieren Verzögerung der Ablegezeit von im Mittel 18 ± 7 min (minimal 4, maximal 40 min). Dies entspricht einer Verzögerung der Eintreffzeit der Seenotrettungsboote um 86 % bzw. der Seenotrettungskreuzer um 51 % von der mittleren Gesamteintreffzeit, weil (meist) kein Arzt für das Einsatzmittel unmittelbar verfügbar war. Die Einsatzindikationen mit Wartezeit auf einen anrückenden Notarzt waren im betrachteten Zeitraum u. a. mehrfach Myokardinfarkte, Atemnot, Schlaganfälle, schwere Traumata, eine Reanimation sowie ein erkrankter Säugling. Hierzu sei angemerkt, dass das Personal in der Seenotrettung bei bestehender Indikation in der Regel auf den Notarzt warten muss und nicht „vorfahren“ kann, einerseits aufgrund heterogener Ausbildung und andererseits, da ein späteres Nachführen im Sinne eines Rendezvous-Verfahrens schon allein mangels zusätzlichen Fahrzeugs regelhaft entfällt. In Anbetracht der Einsatzindikationen wäre eine Verzögerung des Ausrückens des einzigen geeigneten Rettungsmittels im Landrettungsdienst aufgrund von systematischem Nichtvorhandensein eines Notarztes um im Mittel 18 min, insbesondere in Anbetracht der mittleren zusätzlich benötigten Zeit von 21 min (Seenotrettungsboote) und 35 min (Seenotrettungskreuzer) bis zum Eintreffen am Patienten, undenkbar.

Die Analyse der Einsatzfallschwere auf Basis des NACA-Scores gibt Hinweise darauf, dass bei medizinischen Einsätzen der DGzRS regelhaft ärztliches Personal nötig ist. In der Gesamtheit aller Einsätze fanden sich 38 % Einsätze mit einer Erkrankungsschwere von NACA 4 und 31 % mit einer Erkrankungsschwere von NACA 5 oder schwerer (Median des NACA-Scores: 4 mit und ohne Patienten im Wasser). Es bestand also in 69 % aller medizinischen Einsätze (mit oder ohne Notarzt) akute Lebensgefahr für den Patienten (Abb. 2). Auch nach Ausschluss der Patienten im Wasser bestand noch für über die Hälfte der behandelten Patienten (54 %) akute Lebensgefahr (Abb. 2). Im Lübecker Notarztdienst bestand lediglich für 46 % der Patienten akute Lebensgefahr (Median des NACA-Scores: 3 (3; 4)) (Abb. 3). Dies sind deutlich weniger lebensgefährlich Erkrankte, als in der Seenotrettung (Median des NACA-Scores 4 (DGzRS) vs. 3 (NEF): *p* < 0,0001 (bei allen Einsätzen der DGzRS) bzw. 4 (DGzRS) vs. 3 (NEF) *p* = 0,0018 (bei den Einsätzen der DGzRS, exklusive solcher mit Patienten im Wasser)). Dies ist beachtlich, da in vielen Einsätzen der Seenotretter kein Notarzt anwesend war und auch die auf dem Rettungswagen vorgeschriebene medizinische, nichtärztliche Qualifikation des Notfallsanitäters, bei den mit der Situation konfrontierten Seenotrettern nur in Ausnahmefällen vorausgesetzt werden kann.

Das große Ausmaß schwer erkrankter Patienten zeigt sich zusätzlich zu dem erhobenen NACA-Score darin, dass 88 % der Patienten an den Landrettungsdienst übergeben und 6 % durch RTH transportiert wurden. Ein Krankenhaustransport der Patienten war also in 94 % der Fälle nötig. Trotz der in jedem Fall stattgefundenen Vorsichtung der Patienten durch medizinisches Personal wurde in 24 % die Notwendigkeit eines notarztbegleiteten Weitertransports im RTW gesehen.

Die große Zahl internistischer Krankheitsbilder in der Seenotrettung steht im Einklang mit Voruntersuchungen aus Frankreich, die in HEMS-Einsätzen bis 320 km Offshore der französischen Atlantikküste insbesondere internistische Einsatzindikationen fanden, wobei das ACS der häufigste Alarmierungsgrund war und folgend in einer relevanten Anzahl von Einsätzen auch ein STEMI vorlag (11 Patienten mit STEMI bei 36 Alarmierungen wegen eines ACS) [16]. Diese hohe Rate an Patienten mit einem STEMI unter allen Alarmierungen wegen einer ACS-Symptomatik gibt Hinweise auf die hohe durchschnittliche Krankheitsschwere in diesem Patientenkollektiv. Weiterhin gab es im Rahmen medizinischer Einsätze der Seenotretter mit 5,5 % vs. 4,4 % (*p* = 0,46) vergleichbar viele Reanimationen und mit 4,9 % vs. 3,6 % (*p* = 0,31) vergleichbar viele Todesfälle wie im Notarztdienst der Hansestadt Lübeck.

**Figure 6:**
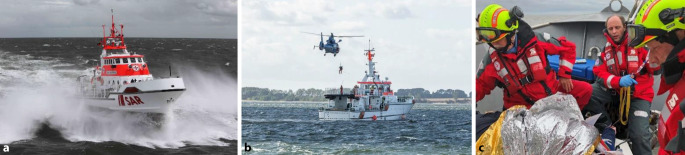

Neben den medizinischen Herausforderungen (die schon fachlich aufgrund der geringen Häufigkeit eine ungewohnte Herausforderung für den Landrettungsdienst darstellen [18]), stellen Einsätze mit der DGzRS häufig auch besondere logistische Ansprüche an eingesetzte Notärzte. Die Versorgung Erkrankter an Bord wird durch Seegang, Enge und Lärm erschwert (Abb. 6). Außerdem muss der Arzt genaue Kenntnis der Ausrüstung haben, da ein nachträgliches Anfordern weiteren Materials nicht möglich ist. Im Einsatzablauf entscheidend ist eine ausreichende Kenntnis der besonderen Ausstattung (z. B. Überlebensanzug), des sicheren Verhaltens an Bord eines Schiffes oder Helikopters und insbesondere auch des sicheren Durchführens eines taktisch u. U. sehr wichtigen Transfers zwischen Schiff und Helikopter (Abb. 6).

All dies kann bei Notärzten der Regelrettung nicht vorausgesetzt werden. Notärzte für medizinische Seenotfälle können daher nicht einfach und ohne Risiko aus dem Regelnotarztdienst herangeführt werden, sondern bedürfen einer grundlegenden Ausbildung in den Besonderheiten des maritimen Umfelds. Anderenfalls besteht die Gefahr, dass der herangeführte Notarzt aufgrund fehlender Ausbildung keine Hilfe für die Besatzung der Rettungseinheit ist, sondern ein Hindernis darstellt und im Extremfall (z. B. bei einem Sturz ins Wasser) weitere Kräfte bindet.

Eine effektivere ärztliche Unterstützung der deutschen Seenotrettung könnte z. B. durch Erweiterung der Einsatzkapazitäten seenotrettungstauglicher Rettungshubschrauber erfolgen. Darüber hinaus sollten die bestehenden telemedizinischen Konzepte (Telenotarzt) ausgebaut werden und deren Effizienz auch für die Seenotrettung in entsprechenden Studien überprüft werden.

## Limitationen

Die vorliegende Untersuchung weist einige Limitationen auf. Zunächst handelt es sich um eine rein retrospektive Arbeit, weshalb auf die Datenerfassung und deren Qualität kein Einfluss genommen werden konnte. Insbesondere die Dokumentationsqualität ist deutlich verbesserungswürdig und die Auswertung der vorliegenden Daten mag deshalb Fehlern unterliegen. Mit einer hochqualitativen Dokumentation von Situation, Messwerten, Maßnahmen und Verlauf ließen sich präzisere Auswertungen durchführen.

Die Einsatzprotokolle sind situativ-deskriptiv; es wurden wenige Messwerte erhoben und wenige medizinische Maßnahmen dokumentiert. Pathophysiologische Variablen standen mithin für eine Auswertung nicht ausreichend zur Verfügung. Um dennoch eine Vergleichbarkeit der Erkrankungsschwere zu ermöglichen, wurde der NACA-Score gewählt, der typischerweise auch durch Beurteilung deskriptiver Erkrankungsszenarien ohne Zuhilfenahme von Messwerten bestimmt werden kann. Dieser NACA-Score wurde nicht von den am Einsatz beteiligten Rettungskräften vergeben, sondern im Nachhinein bestimmt. Die rein retrospektive Bestimmung anhand von Protokollen mit teilweise schwacher Dokumentationsqualität bedingt hier allerdings die Möglichkeit von Fehlannahmen.

Die vorliegende Arbeit beschäftigt sich ausschließlich mit von der DGzRS (als Hauptakteur der deutschen Seenotrettung) durchgeführten Primäreinsätzen. Sie betrachtet nicht, ob und in welchem Umfang mögliche Dritte an Rettungseinsätzen beteiligt sind. Der Vergleich erfolgt orientierend mit den Notarzteinsätzen eines NEF (der Hansestadt Lübeck) mit städtisch geprägtem Einsatzspektrum und nicht gebietskörperschaftsübergreifend; für die Aspekte Alter, Geschlecht und Erkrankungsschwere kann hier ausreichende Repräsentativität vermutet werden, für die gruppierten Einsatzindikationen ist insbesondere das Trauma/Nontrauma-Verhältnis möglicherweise nicht repräsentativ. Eine Ausweitung der Betrachtung auf alle im Bereich der Seenotrettung und Offshoremedizin beteiligten Akteure und ein Vergleich mit einem größeren und heterogeneren Stadt‑/Landrettungskollektiv könnte die Aussagekraft in Folgestudien weiter steigern.

## Zusammenfassung

Die vorliegende Arbeit beschreibt detailliert das medizinische Einsatzspektrum der DGzRS über 2 Jahre. Bei 182 analysierten medizinischen Seenotrettungseinsätzen benötigten die Einsatzeinheiten im Mittel 30 min bis zur Ankunft und 43 min für Behandlung, Transport und Übergabe des Patienten. Die in den Einsätzen behandelten Patienten scheinen den Ergebnissen zufolge jünger und häufiger lebensbedrohlich erkrankt gewesen zu sein, als das Kollektiv der durch Notärzte behandelten Patienten in der Hansestadt Lübeck. Weiterhin kam es vergleichbar häufig zu Reanimationen und Todesfällen wie bei den Notarzteinsätzen des Vergleichsnotarzteinsatzfahrzeuges. Trotz der größeren Erkrankungsschwere konnten nur 34,6 % der medizinisch relevanten Seenotrettungseinsätze durch einen Notarzt begleitet werden. In ca. der Hälfte der 44 Fälle mit der Notwendigkeit eines von extern anrückenden Notarztes kam es zu einer Verzögerung des Ablegens von im Mittel 18 min. Die Einsatzindikation war hier meist dringlich und ließ eine Verschlechterung des Patienten-Outcome durch verzögerte Therapie erwarten. Die Untersuchung gibt Hinweise darauf, dass medizinische Hilfe regelmäßig und trotz zeitkritischer Indikation aufgrund unzureichend vorgehaltenen ärztlichen Personals erst verzögert eintrifft. Aufgrund der besonderen Anforderungen des maritimen Umfelds ist eine Besetzung der Rettungseinheiten der DGzRS durch nicht spezifisch ausgebildete Ärzte der Regelrettung als systematisches Konzept nicht geeignet. Die aktuell vorgehaltene Kapazität an geeigneten Notärzten (und telenotärztlicher Unterstützung) sollte ausgebaut werden.

## Fazit

Es gibt dringenden Bedarf an einer effektiveren Unterstützung der DGzRS durch für den maritimen Einsatz ausreichend ausgebildetes ärztliches Personal.
